# ACSL4, PUFA, and ferroptosis: new arsenal in anti-tumor immunity

**DOI:** 10.1038/s41392-022-01004-z

**Published:** 2022-04-22

**Authors:** Boyi Gan

**Affiliations:** Department of Experimental Radiation Oncology, The University of Texas MD Anderson Cancer Center, Houston, TX USA

**Keywords:** Tumour immunology, Cell biology, Cancer therapy

In a recent study published in *Cancer Cell*, Liao et al.^1^ identified CD8^+^ T-cell-derived interferon (IFN)γ in combination with polyunsaturated fatty acids (PUFAs) as a natural ferroptosis inducer (FIN) to trigger tumor ferroptosis and promote anti-tumor immunity in an acyl-coenzyme A synthetase long-chain family member 4 (ACSL4)-dependent manner.

**Fig. 1:**
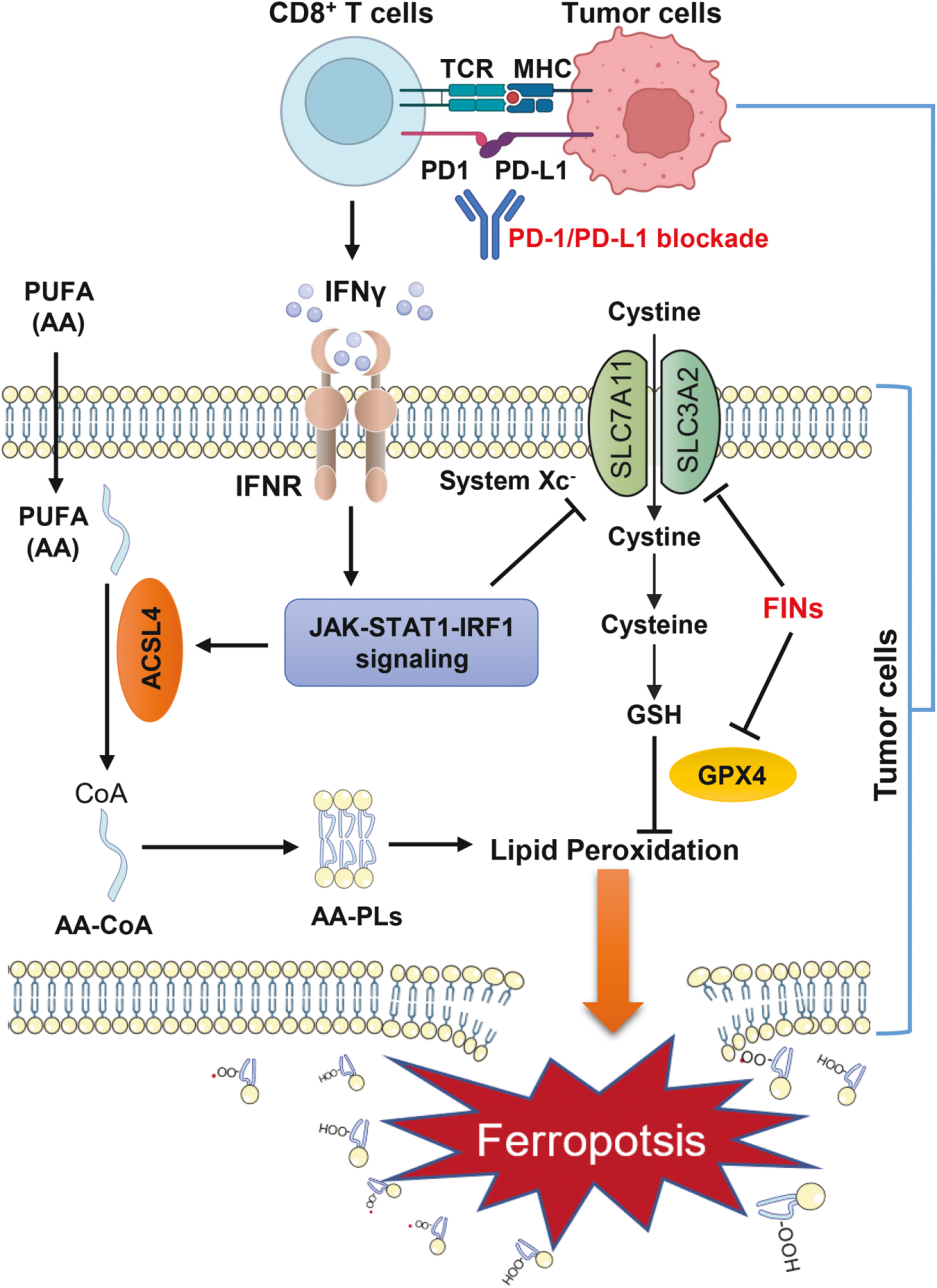
PUFAs and CD8^+^ T-cell-derived IFNγ act in concert to induce tumor ferroptosis and mediate anti-tumor immunity. PUFA-PLs in the cellular membrane are susceptible to peroxidation, and excessive lipid peroxidation leads to membrane rupture and ferroptosis. Lipid peroxidation is kept in check by ferroptosis defense mechanisms mediated by GSH-GPX4 signaling, wherein GPX4 utilizes GSH to neutralize lipid peroxides, and cystine uptake mediated by system Xc^–^ (which consists of SLC7A11 and SLC3A2) provides the key precursor cysteine for GSH synthesis. Blocking system Xc^–^ or inactivating GPX4 by FINs curbs ferroptosis defense and induces ferroptosis in tumor cells. IFNγ secreted by CD8^+^ T cells binds to IFNR, activates the JAK-STAT1-IRF1 signaling axis, and induces ACSL4 expression in tumor cells. The increase in ACSL4 expression, in combination with AA supplementation, promotes AA containing-PL synthesis. In addition, IFNγ reduces SLC7A11 expression and weakens ferroptosis defense in tumors cells. These two effects caused by combined treatment with IFNγ and PUFAs (namely increased AA-PL synthesis and weakened ferroptosis defense) induce potent ferroptosis in tumor cells. Consequently, PUFA treatment enhances anti-tumor immunity and its combination with immune checkpoint blockade therapies (such as PD-1/PD-L1 blockade) exerts potent tumor suppressive effects. Figure adapted from images created in Biorender. AA arachidonic acid, ACSL4 acyl-coenzyme A synthetase long-chain family member 4, CoA coenzyme A, FIN ferroptosis inducer, GPX4 glutathione peroxidase 4, GSH reduced glutathione, IFNγ interferon γ, IFNR interferon γ receptor, IRF1 interferon regulatory factor 1, JAK janus kinase, MHC major histocompatibility complex, PD-1 programmed cell death protein 1, PD-L1: programmed death-ligand 1, PL phospholipid, PUFA polyunsaturated fatty acid, SLC3A2 solute carrier family 3 member 2, SLC7A11 solute carrier family 7 member 11, STAT1 signal transducer and activator of transcription 1, TCR T-cell receptor.

Ferroptosis refers to a form of regulated cell death triggered by excessive iron-dependent peroxidation of PUFA-containing phospholipids (PUFA-PLs) in the cellular membrane.^2^ Correspondingly, ferroptosis can be blocked by inactivation of enzymes involved in PUFA-PL biosynthesis, such as ACSL4, which ligates free PUFAs with coenzyme A to produce PUFA-CoAs for their subsequent incorporation into PLs (Fig. 1).^2^ Although PUFAs are required for ferroptosis onset, PUFA treatment alone typically is not sufficient to induce potent ferroptosis in cell lines. This is partly because cells are also equipped with strong ferroptosis defense mechanisms, including glutathione peroxidase 4 (GPX4)-dependent anti-oxidant systems, wherein GPX4 uses glutathione as its co-factor to detoxify lipid peroxides and suppress ferroptosis, whereas system Xc^–^ (which consists of the transporter subunit SLC7A11 and the regulatory subunit SLC3A2) imports cystine for glutathione synthesis and ferroptosis defense (Fig. 1).^2^

Notably, the widely used FINs (synthetic compounds that are capable of inducing potent ferroptosis in cell lines) operate by curbing ferroptosis defense mechanisms. For example, RSL3 is a synthetic compound that induces ferroptosis by inactivating GPX4.^2^ Although FINs provide great tools for ferroptosis research, the initial reliance on these compounds to study ferroptosis has raised the concern of whether ferroptosis simply reflects an artificial cell death modality that only occurs in cell cultures treated with synthetic compounds. In recent years, it has been increasingly appreciated that ferroptosis can occur in diverse pathological conditions such as cancer and degenerative diseases;^2^ however, natural ferroptosis inducing conditions still remain largely elusive.

Recent studies revealed that common cancer therapies, such as radiotherapy, are capable of inducing ferroptosis.^3,4^ Elegant studies from Zou and colleagues^4,5^ also showed that immunotherapy can trigger tumor ferroptosis. They found that IFNγ secreted by CD8^+^ cytotoxic T cells sensitize tumor cells to FIN-induced ferroptosis partly by suppressing the expression of SLC7A11 (Fig. 1); however, IFNγ treatment alone fails to induce obvious ferroptosis in tumor cells.^4,5^ Reasoning that IFNγ likely needs to cooperate with other factors to induce ferroptosis and considering the intimate link between ferroptosis and lipid metabolism, in this study the authors first tested whether IFNγ can synergize with any fatty acid to trigger ferroptosis in tumor cells. These analyses revealed that while treatment with arachidonic acid (AA, a 20-carbon chain PUFA with four double bonds; C20:4) alone triggers little ferroptosis, AA synergizes with IFNγ to induce potent ferroptosis in diverse cancer cell lines in an ACSL4-dependent manner.^1^

Mechanistic studies revealed that IFNγ induces ACSL4 expression through the Janus kinase (JAK)-signal transducer and activator of transcription 1 (STAT1)-interferon regulatory factor 1 (IRF1) signaling axis: IFNγ binds to IFN receptor and activates JAK-STAT1 signaling, resulting in IRF1 binding to IFN-stimulated response element located on the *ACSL4* promoter and upregulation of *ACSL4* transcription (Fig. 1).^1^ Lipidomics analyses suggested that, under IFNγ + AA combination treatment conditions, the increased levels of both AA and ACSL4 significantly promote AA incorporation into PLs.^1^ Consequently, the increased PUFA-PL synthesis, coupled with weakened ferroptosis defense due to decreased SLC7A11 expression in tumor cells caused by IFNγ treatment, induces potent tumor cell ferroptosis (Fig. 1).

This prompted the authors to further examine whether ACSL4 is engaged in CD8^+^ T-cell-mediated anti-tumor immunity. They found that *Acsl4* deletion does not affect xenograft tumor growth in immunodeficient mice, but significantly promotes tumor growth in immunocompetent mice.^1^ Furthermore, the combination treatment of low-dose AA and anti-PD-L1 reduced tumor growth more potently than either treatment alone; notably, AA treatment did not affect xenograft tumor growth in immunodeficient mice.^1^ Therefore, at least in the preclinical models tested in this study, the effects of both ACSL4 ablation and AA treatment on tumor growth (to promote or suppress tumor growth, respectively) appear to depend on intact immune systems. Future investigations should be directed toward further evaluating this in relevant genetically engineered mouse models.

Overall, this study identifies IFNγ (secreted by CD8^+^ T cells) in combination with AA (taken up from the tumor microenvironment) as a novel natural FIN, and suggests that ACSL4-mediated PUFA-PL synthesis and ferroptosis execution have an important role in anti-tumor immunity.^1^ This study also raises several intriguing questions for future investigations. On the fundamental level, ferroptosis induced by IFNγ + AA combination exhibits some unique features distinctive from that induced by classic FINs (such as RSL3). For example, it is known that while PUFAs (fatty acids that contain more than one double bond) are required for RSL3-induced ferroptosis, another class of fatty acids called monounsaturated fatty acids (MUFAs, which contain one double bond) actually have a suppressive effect on RSL3-induced ferroptosis.^2^ This is because lipid peroxidation reactions require bis-allylic moieties, which exist in PUFAs but not in MUFAs. Instead, MUFAs suppress lipid peroxidation and ferroptosis likely by competing with PUFAs for their incorporation into PLs.^2^ Interestingly, this study found that MUFAs such as oleic acids (C18:1), similar to PUFAs, synergize with IFNγ to promote ferroptosis in an ACSL4-dependent manner. It should be noted that, because mammalian cells lack certain desaturases, oleic acid cannot be converted to AA (though desaturation and elongation, as occurs in plant cells), and AA must be obtained from diet or medium in mammalian cells. Therefore, how oleic acids in the context of IFNγ treatment can promote tumor cell ferroptosis remains unclear and will be an interesting topic for future studies. On the translational level, this study indicates that AA (supplied to patients as a diet) in combination with immune checkpoint blockades could be a novel and effective therapeutic strategy in cancer treatment.^1^ It will be important to identify patient populations that might be suitable for this combination therapy (such as guided by ACSL4 expression) and to further test this therapy in future clinical trials.
